# Mandatory role of proteinase-activated receptor 1 in experimental bladder inflammation

**DOI:** 10.1186/1472-6793-7-4

**Published:** 2007-03-30

**Authors:** Ricardo Saban, Michael R D'Andrea, Patricia Andrade-Gordon, Claudia K Derian, Igor Dozmorov, Michael A Ihnat, Robert E Hurst, Carole A Davis, Cindy Simpson, Marcia R Saban

**Affiliations:** 1 Department of Physiology, The University Oklahoma Health Sciences Center, Oklahoma City, OK 73104, USA; 2 J&J Pharmaceutical Research and Development Spring House, PA 19477-0776, USA; 3 Oklahoma Medical Research Foundation (OMRF), Arthritis and Immunology Research Program, Microarray/Euk. Genomics Core Facility, Oklahoma City, Oklahoma 73104, USA; 4 Department of Cell Biology, The University Oklahoma Health Sciences Center, Oklahoma City, OK 73104, USA; 5 Department of Urology, The University Oklahoma Health Sciences Center, Oklahoma City, OK 73104, USA

## Abstract

**Background:**

In general, inflammation plays a role in most bladder pathologies and represents a defense reaction to injury that often times is two edged. In particular, bladder neurogenic inflammation involves the participation of mast cells and sensory nerves. Increased mast cell numbers and tryptase release represent one of the prevalent etiologic theories for interstitial cystitis and other urinary bladder inflammatory conditions. The activity of mast cell-derived tryptase as well as thrombin is significantly increased during inflammation. Those enzymes activate specific G-protein coupled proteinase-activated receptors (PAR)s.

Four PARs have been cloned so far, and not only are all four receptors highly expressed in different cell types of the mouse urinary bladder, but their expression is altered during experimental bladder inflammation. We hypothesize that PARs may link mast cell-derived proteases to bladder inflammation and, therefore, play a fundamental role in the pathogenesis of cystitis.

**Results:**

Here, we demonstrate that in addition to the mouse urinary bladder, all four PA receptors are also expressed in the J82 human urothelial cell line. Intravesical administration of PAR-activating peptides in mice leads to an inflammatory reaction characterized by edema and granulocyte infiltration. Moreover, the inflammatory response to intravesical instillation of known pro-inflammatory stimuli such as E. coli lipopolysaccharide (LPS), substance P, and antigen was strongly attenuated by PAR1-, and to a lesser extent, by PAR2-deficiency.

**Conclusion:**

Our results reveal an overriding participation of PAR1 in bladder inflammation, provide a working model for the involvement of downstream signaling, and evoke testable hypotheses regarding the role of PARs in bladder inflammation. It remains to be determined whether or not mechanisms targeting PAR1 gene silencing or PAR1 blockade will ameliorate the clinical manifestations of cystitis.

## Background

In general, inflammation plays a role in most bladder pathologies, including bladder cancer [1-4] and represents a defensive reaction to injury caused by physical damage, chemical substances, micro-organisms, or other agents [1,2]. In particular, bladder neurogenic inflammation involves the participation of mast cells and sensory nerves. Evidence for a role of mast cells in cystitis was reviewed recently [5] and includes the presence of mast cells containing tryptase in the bladder [6] and urine of IC patients [6,7], and that mast cell counts in IC patients are one of the few features significantly associated with night-time frequency of urination [8]. We have previously presented direct evidence indicating a key role for mast cells and their products in bladder inflammation [9-11], and others emphasized the role of mast cell products in bladder disorders [12-14].

As a consequence of inflammation, products of mast cell degranulation, such as tryptase, can be found in the urine of cystitis patients [15]. In addition to tryptase, other serine proteases such as thrombin and trypsin are produced during tissue damage and make important contributions to tissue responses to injury, repair, cell survival, inflammation [16-19], and pain [20-24]. Tissue responses to these enzymes are modulated by protease-activated receptors (PARs), a unique class of G protein-coupled receptors that use a fascinating mechanism to convert an extracellular proteolytic cleavage event into a trans-membrane signal. These receptors carry their own ligands, which remain cryptic until unmasked by receptor cleavage (for a review, please see references [20,23,25,26]).

Four PARs have been cloned so far, and all four PARs are co-expressed in the mouse bladder urothelium [27], with PAR2 and PAR3 being the most abundant in the bladder epithelial layer. Although information regarding the presence of PARs in human urothelial cells is scanty, indirect evidence indicates that human cancer urothelial cells, such as J82, augment the conversion of prothrombin to thrombin, a key activator of PARs [28]. In addition, thrombin and other elements of the coagulation cascade activate J82 carcinoma cells, inducing Ca^2+ ^mobilization, phospholipase C activity, and cell migration [29]. Another human bladder cell line, RT4, responds to thrombin, tryptase or PAR-APs with an increase in intracellular phospholipase A_2 _activity, arachidonic acid and prostaglandin E_2 _release [30]. In this work, we determined whether all four receptors are present in the human urothelial J82 cell line.

In addition to the urothelium, PAR1 and PAR2 are also expressed in mouse detrusor muscle, and PAR4 is expressed in mouse peripheral nerves and plexus cell bodies [27]. Similarly, in rats PAR2, 3, and 4 are expressed in urothelium, detrusor muscle, and bladder nerve fibers, and bladder afferent cells in dorsal root ganglia express PAR2 to 4 [31]. Confocal microscopy has revealed the co-localization of PAR2, 3, and 4 with protein gene products 9.5 and vanilloid receptor 1, suggesting that PARs are distributed in C-fiber bladder nerves [31]. In addition, PARs are differentially modulated during mouse bladder inflammation. Urothelial PAR2 and, to a lesser extent, PAR1 are down-regulated in acute inflammation whereas PAR3 and PAR4 are up-regulated [27]. Bladder fibroblasts were found to present a clear demarcation in PAR expression in response to acute and chronic inflammation [27]. Additional evidence for the participation of PARs in the bladder inflammatory response was the finding that known pro-inflammatory stimuli such as LPS, substance P, and antigen challenge induce an increase in PAR4 RNA within four hours [32]. Upregulation of PAR protein levels have been shown to be part of rat bladder responses to cyclophosphamide [31].

In order to better understand the role of PARs in cystitis, we used a well-established mouse model [1,2,26] to determine the relative potency of PAR-activating peptides (PAR-APs). PAR3-AP was not included in this research because of a lack of specificity of the available reagent. Comparison of inflammatory responses in wild type, PAR1- and PAR2-deficient mice, revealed a mandatory role of PAR1 and, to a lesser extent, PAR2 in mediating bladder responses to a variety of pro-inflammatory stimuli.

The combination of morphological results reveals an overriding participation of PAR1 receptors in bladder inflammation and provides a working model to investigate the inflammatory cascade downstream of PAR activation.

## Results

### Human urothelial cell line expresses all four PARs

**Figure 1:**
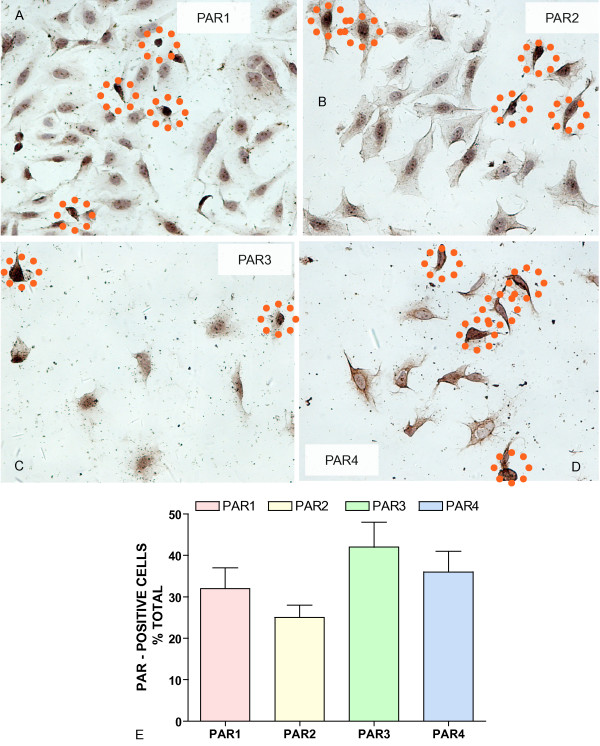
**A-E**. PAR immunohistochemistry in J82 human urothelial carcinoma cell line. Representative photomicrograph obtained in J82 cell line stained with PAR specific antibodies. J82 cells were fixed and incubated with primary polyclonal (Santa Cruz Biotechnology, Santa Cruz, CA) antibodies: A = PAR-1 (1:20), B = PAR-2 (1:100), C = PAR-3 (1:5), and D = PAR-4 (1:50). Slides were washed and incubated with biotinylated secondary antibodies (Vector Labs), goat anti-rabbit (polyclonal antibodies). Orange dotted circles highlight some cells considered positives for the particular receptor. Original magnification was ×200. Figure 1E represents the average and SEM of number of PAR-positive cells as percent of the total cells per field.

**Figure 2:**
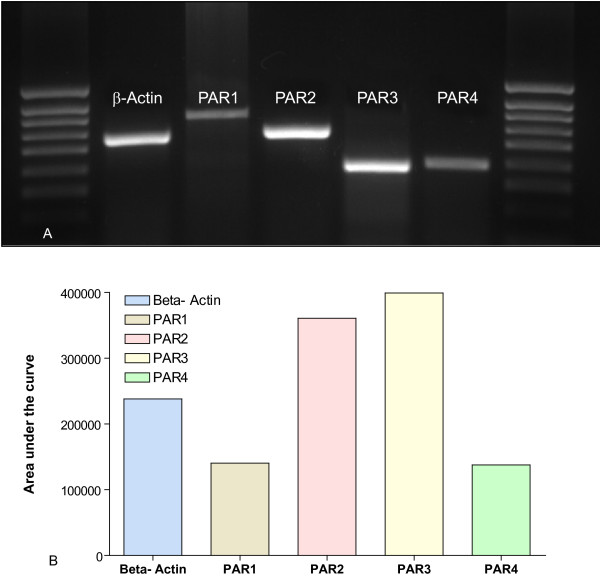
**A-B**. Polymerase Chain Reaction for detection of PARs message in J82 human urothelial carcinoma cell line. Figure 2A is a photomicrograph of the gel and Figure 2B represents the area under the curve as quantified using Image J software [74]. Primers used in this experiment are described in additional file 1 (Table 1).

In order to extend our previous results obtained with the mouse bladder [27], we also tested the presence of PA receptors in the J82 human urothelial cancer cell line. Our results indicate all four PARs are detectable at the protein level by immunohistochemistry (Figure 1A–E) and at the message level by polymerase chain reaction (Figure 2A–B) in human urothelial cancer cell line.

### PAR-APs induce bladder inflammation

**Figure 3:**
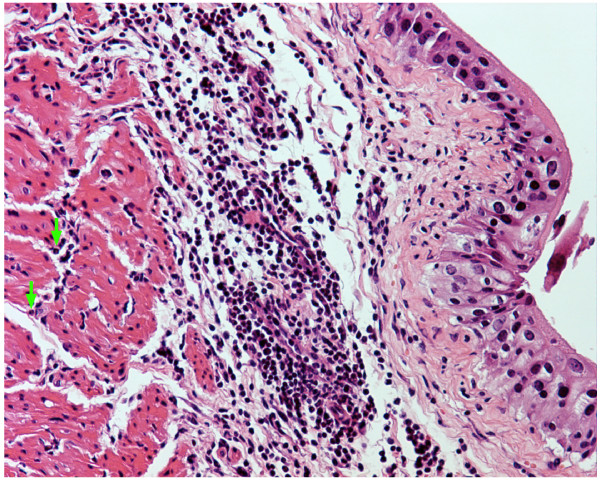
**PAR4-AP induces bladder inflammation**. Anesthetized female C57BL6 mice were catheterized, the bladder was emptied, and a volume of 200 μl of a solution of PAR4-AP (10 μM) was instilled into the urinary bladder. Twenty four hours later, bladders were removed, processed for histology, and stained with H&E. At low magnification, photomicrograph illustrates the distribution of inflammatory cells extending from the submucosa to deep regions in the detrusor smooth muscle (green arrows). Magnifications = ×100

**Figure 4:**
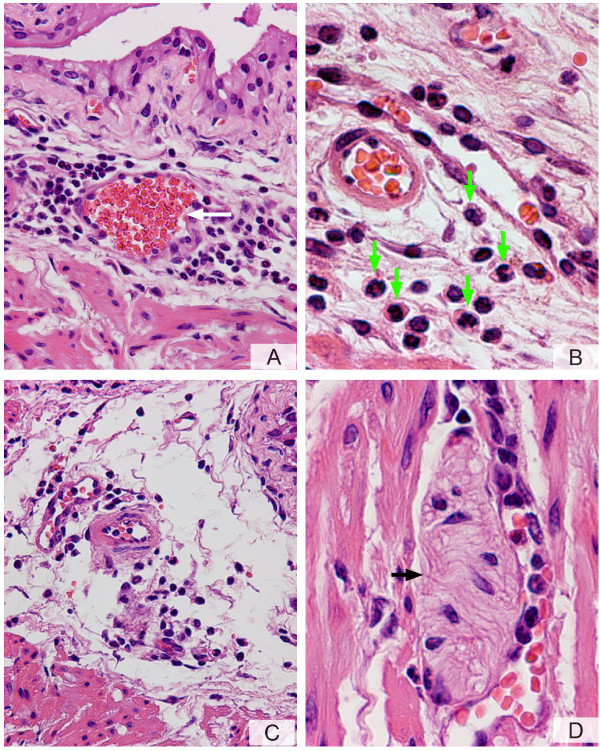
**A-D. PAR4-AP induces bladder inflammation**. Anesthetized female C57BL6 mice were catheterized, the bladder was emptied, and a volume of 200 μl of a solution of PAR4-AP (10 μM) was instilled into the urinary bladder. Twenty four hours later, bladders were removed, processed for histology, and stained with H&E. A characteristic photomicrograph represents sub-urothelium inflammatory infiltrate around a blood vessel (A) and dilation of blood vessels (white arrow). At higher magnification (B), it was possible to visualize that the majority of inflammatory cells in response to PAR4-AP presented a characteristic "doughnut" shape indicative of mouse PMNs (green arrowhead). The submucosal edema is illustrated in C. Figure 4D illustrates inflammatory cells surrounding a structure  resembling a nerve element (black arrow). Magnifications A = ×200, B = ×400, C = ×200, and D = ×400.

Next, we determined whether activation of PARs in mice would induce bladder inflammation. Figure 3 shows a representative photomicrograph demonstrating that an inflammatory response was mounted twenty-four hours after bladder instillation with PAR4-AP (10 μM). Inflammation secondary to PAR4-AP was characterized by vasodilation (Figure 4A), sub-epithelial infiltration of inflammatory cells (Figure 4B), and edema (Figure 4C). A high magnification photomicrograph (Figure 4D) indicates that the majority of inflammatory cells responding to PAR4-AP were polymorphonuclear [PMNs] leukocytes characteristic of acute bladder inflammation [32]. In addition, the cellular infiltrate expanded from submucosal layers towards the detrusor smooth muscle (green arrows in figure 3A) and within the detrusor nerve elements, previously shown to contain PAR4 (figure 9 of the reference [27]), are surrounded by inflammatory cells. Similar results were obtained 48 hours after bladder instillation with PAR4-AP with the addition of macrophages to the lesion (data not shown).

**Figure 5:**
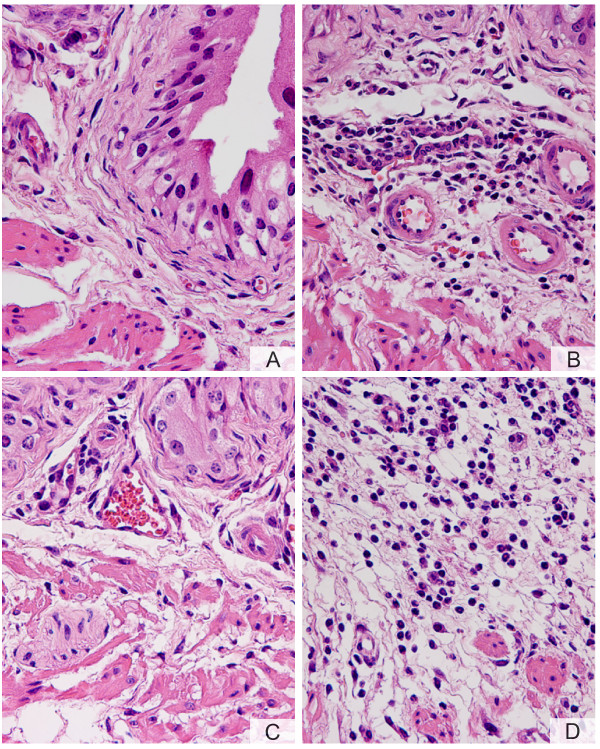
**A-D. Comparison of different PAR-APs in inducing mouse bladder inflammation**. Characteristic photomicrographs of bladders removed from female C57BL6 mice that received instillation of (A = control peptide; B = PAR1-AP; C = PAR2-AP, and D = PAR4-AP). All peptides were at the concentration of 10 μM and bladders were removed 24 hours after challenge. Of note, absence of inflammation following instillation with control peptide (A), submucosal PMN infiltrate induced by PAR1-AP (B), modest perivascular infiltration in response to PAR2-AP (C), and overwhelming PMN infiltration in response to PAR4-AP (D). Magnifications: A, B, C, and D = ×200.

**Figure 6:**
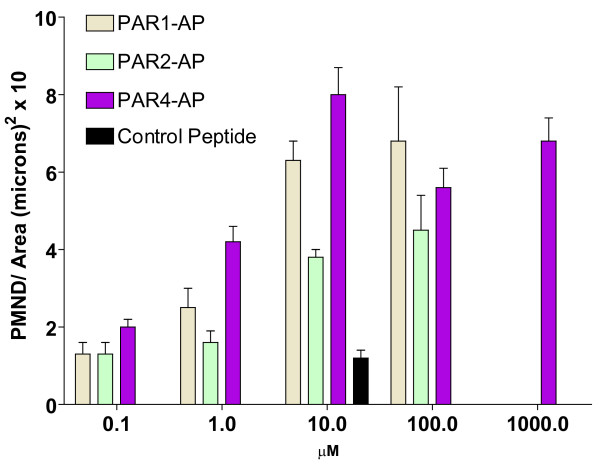
**Effect of different concentrations of PAR1-, PAR2-, and PAR4-AP on PMNs migration**. Anesthetized female C57BL6 mice were catheterized, the bladder was emptied, and a volume of 200 μl of a solution of control peptide (10 μM) or PAR1-, PAR2-, and PAR4-AP at different concentrations (0.1 to 1000 μM) was instilled into the urinary bladder. The number of PMNs migration into the mouse bladders was examined 24 hours after instillation (n = 8 for PAR4-AP and control peptide and n = 4 for PAR1-AP and PAR2-AP).

Next, we compared the effect of 10 μM concentrations of PAR1-AP, PAR2-AP, and PAR-4-AP on the degree of PMN infiltration into the urinary bladder. The representative photomicrographs on figure 4 indicate that contrast to the control peptide which failed to induce bladder inflammation (Figure 5A), PAR1- (Figure 5B), PAR2- (Figure 5C), and PAR4-AP (Figure 5D) induced different densities of inflammatory cell infiltrate. Figure 6 summarizes the results obtained with different concentrations of PAR-APs. An inflammatory cell infiltrate was detected at concentrations as low as 0.1 μM and reached a peak at 10 μM PAR4-AP. These results indicate that, at least in the mouse bladder, the instillation of 10 μM PAR4-AP or 100 μM PAR1-AP induces a strong inflammatory reaction characterized by the highest number of PMNs infiltrating the bladder. Both PAR1-AP and PAR2-AP at 10 μM concentration induced bladder inflammation but the number of PMNs that infiltrated the urinary bladder was smaller than that observed with PAR4- AP (10 μM). No inflammation was observed in mice instilled with pyrogen-free saline or 10 μM control peptide dissolved in PBS.

**Figure 7:**
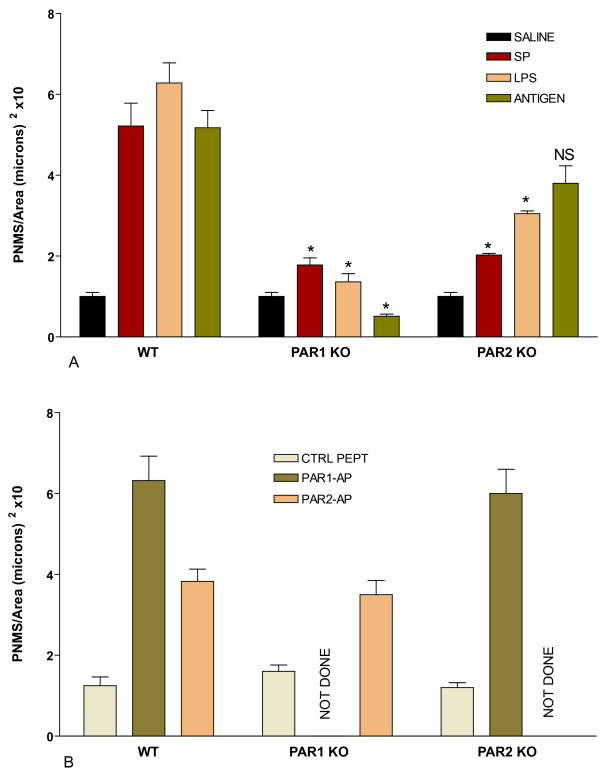
**A-B. **Comparison of the inflammatory responses (PMNs infiltration) obtained with wild type (C57BL/6J), PAR1^-/-^, and PAR2^-/- ^mice. Responses were obtained 24 hours after bladder instillation with one of the listed substances. **Figure 7A **represents the response to substance P (10μM), Escherichia coli LPS strain 055:B5 (100 μg/ml), and DNP4-OVA (in sensitized mice; 1 μg/ml) compared to saline (black bars). **Figure 7B **represents the responses to PAR1-AP (10 μM) and PAR2-AP (10 μM) compared to control inactive peptide (10 μM). Number of mice = 6–8 per group. NS= not significant. Asterisks represent a statistically significant difference (p < 0.05) between the responses obtained with each pro-inflammatory stimuli in PAR1^-/- ^or PAR2^-/- ^and the respective response obtained in wild type mice.

PAR1^-/- ^and PAR2^-/- ^mice were used to determine whether those receptors were downstream of a common inflammatory cascade. We chose the following classical inflammatory stimuli: substance P, LPS, and antigen-challenge (in sensitized mice) because they were shown to depend on mast cells for eliciting bladder inflammation [7,8,27], and products of mast cell degranulation such as chymase and tryptase activate PAR1 [33,34] and PAR2 receptors [35,36], respectively. For this purpose, groups of anesthetized female, wild type (C57BL/6J), PAR1^-/-^, and PAR2^-/- ^mice (n = 6–8) were instilled with saline, or with control inactive peptide (10 μM), or one of the following substances at concentrations known to induce inflammatory response [32]: substance P (10 μM), Escherichia coli LPS strain 055:B5 (100 μg/ml), DNP4-OVA (in sensitized mice; 1 μg/ml), PAR1-AP (10 μM), and PAR2-AP (10 μM). Twenty-four hours later, bladders were removed for quantification of PMN infiltration. Figures 7A and 7B summarize the results obtained. PAR1^-/- ^mice presented a reduced response to substance P, LPS, and antigen stimulation (Figure 7A). However, the responses to PAR2-AP obtained in PAR1^-/- ^mice were not altered (Figure 7B). The major difference between PAR1^-/- ^and PAR2^-/- ^mice is that the latter still presented an inflammatory response to antigen (Figure 7A).

Regarding other inflammatory cells, bladders isolated from PAR1^-/- ^and PAR2^-/- ^presented equivalent number of mast cells when compared to wild type mice (mast cell/cross section: PAR1^-/- ^= 9.7 ± 1.1; PAR2^-/- ^= 11.7 ± 1.9; wild type = 8.3 ± 0.6 ; *p *values = 0.30 when comparing WT to PAR1^-/- ^and *p= *0.10 when comparing WT to PAR2^-/- ^animals). After stimulation with the pro-inflammatory stimuli, the bladder of wild type mice presented signs of mast cell activation by the presence of degranulation. In contrast, except for some perivascular infiltrate, few inflammatory cells were seen in the bladder of PAR1^-/- ^mice in response to pro-inflammatory stimuli (data not shown). In addition, when mast cells were visible, there was no apparent sign of degranulation, which may reflect poor activation of those cells (data not shown).

These results indicate that PAR1 receptors are essential for bladder inflammation secondary to several pro-inflammatory stimuli, while PAR-2 receptors seem to have a decreased role, at least in mediating the responses to antigen challenge. Because the transgenic mouse models used have been reported to be complete knockouts [18,37-42] and PAR2-AP has no effect on PAR2 knockout mice [43,44], we did not test PAR1-AP in PAR1^-/-^and PAR2-AP in PAR2 ^-/- ^mice.

## Discussion

### Role of PAR in bladder inflammation

Proteases that are released during inflammation and injury cleave PARs on the urothelium, detrusor muscle, and nerve elements to cause neurogenic bladder inflammation [45,46] and hyperalgesia [21,23]. As we demonstrated earlier by IHC, PARs are over-expressed in the bladder following cystitis induced by classical mediators such as LPS and SP [27].

This study determined whether all four PAR receptors are expressed in human urothelial cell line. The presence of multiple PARs on the same cell is thought to extend the range of proteases a cell responds to rather than expand the range of intracellular responses [30]. Human transitional-cell carcinoma (J82) cells express various G protein-coupled receptors, and the presence of PARs in this cell line was based on the effects of thrombin, which induces a strong migration of J82 cells by a mechanism that involves Ca^2+ ^mobilization and activation of Rho kinase leading to a reorganization of the cytoskeleton [29]. In addition to the J82 cell line, expression of PARs in RT4 bladder papilloma cells was inferred based on findings that RT4 cells respond to activators of PARs such as thrombin, tryptase, and PAR-activating peptides[30]. Those PAR activators induced a calcium independent phospholipase A_2 _with the consequent release of arachidonic acid and synthesis of prostaglandin E_2 _[30]. Since the presence of PARs in human urothelium was only based on circumstantial evidence, we determined whether J82 carcinoma cell line expresses PAR protein and RNA. Our results demonstrate that the J82 human urothelial cell line expresses all four PA receptors.

An additional goal of this paper was to determine whether PA receptors are part of the inflammatory cascade mediating the response of the urinary bladder to known pro-inflammatory stimuli. The stimuli were chosen because we have published evidence that each one of them when instilled into the mouse bladder produces inflammation: substance P [47], LPS [48], and antigen challenge of sensitized mice [10,11]. However, the present manuscript did not determine which endogenous substance is responsible for activation of PAR receptors, and therefore, the following discussion is based on the literature and not in the results of this manuscript. The information regarding endogenous activators of PARs in the mouse bladder is, unfortunately, scanty. While human connective tissue mast cells contain the enzymes chymase and tryptase, mice contain numerous related proteases [49-51]. Mouse mast cell protease-7 is a tryptase predominantly expressed in differentiated connective tissue-type mast cells [52]. Mast cell proteases mcpt5 (chymase), mcpt6 and 7 (tryptases) are expressed during the development of the mouse embryo [49]. However, to the best of our knowledge, there is no information regarding which particular tryptase is expressed in the mouse bladder and/or upregulated in this mouse model during inflammation. Although thrombin is a recognized physiological activator of PAR1 and PAR4, the endogenous enzymes responsible for activating PAR2 in urinary bladder are not known. Recently, it was demonstrated that the tissue kallikrein family of proteinases are able to regulate PAR signaling and may represent important endogenous regulators PAR1, PAR2, and PAR4 [53]. The latter are likely to be confirmed in the urinary tract, since members of the kallikrein family play a fundamental role in bladder physiology [54].

Administration of PAR peptide agonists into the urinary bladder of mice elicited an inflammatory reaction characterized by edema and granulocyte infiltration. Interestingly, the inflammatory responses to PAR4-AP peaked at 10 μM and higher concentrations of this peptide (100 μM) failed to produce an additional increase in PMNs migration, but rather induced a lesser degree of inflammation. Several possibilities could explain this finding, including PAR desensitization and endocytosis [55], shedding of these receptors from the cells, as is the case of PAR1 in endothelial cells exposed to thrombin [56], and shedding of urothelial cells bearing these receptor, as we suggested [27]. Although we observed a potent inflammatory response secondary to PAR4-AP, PAR4^-/- ^mice were not available in our laboratory at the time this project started. Nevertheless, our results emphasize the importance of this receptor in cystitis.

In the present work, we determined that PAR1^-/- ^and, to a lesser extent, PAR2^-/- ^mice present decreased inflammatory responses to pro-inflammatory stimuli. The results obtained with PAR-deficient mice provide evidence indicating that substances known to stimulate mast cells, such as SP and antigen, induce a bladder inflammatory reaction that depends on PAR activation. In addition, these results indicate that toxins such as LPS, known to be released during bladder infection, also share the same PAR pathway.

The authors acknowledge the lack of functional data on *in vivo *urinary bladder function at this time. *In vitro *experiments indicate that both trypsin and PAR-2 activating peptide (SLIGRL-NH(2)) produced a concentration-dependent contractile response in the rat urinary bladder preparations. These contractions were abolished by removal of the urinary bladder mucosa and were significantly reduced by the non-steroidal anti-inflammatory drug indomethacin [57]. The release of prostaglandins by PAR-2 activators seems to be partly mediated by the phospholipase A_2 _(iPLA2) [58]. These responses were enhanced in bladders isolated from cyclophosphamide-treated rats [45]. The *in vitro *work confirms the evidence that PAR activation leads to iPLA2-mediated prostaglandin release in human urothelial carcinoma cell line RT4 [30], human bladder microvascular endothelial [59,60], and normal urothelial cells [61]. However, there is no *in vivo *functional data on the effect of PAR stimulation on bladder urodynamic behavior.

It is not clear why the experimental cystitis in PAR2^-/- ^mice was not totally abolished, as seen in PAR1^-/-^. Both PAR1 ^-/- ^and PAR2 ^-/- ^mice have been well characterized [18,37-42]; see [62,63] for reviews. In addition, PAR2-AP has no effect on PAR2 knockout mice [43,44]. Nevertheless, compensatory mechanisms from other PA receptors could be possible. Regarding the difference of response between PAR1 ^-/- ^and PAR2 ^-/- ^mice, this may reflect a different role of the endogenous ligand since PAR1 is activated by thrombin, while PAR2 is activated by mast cell tryptase [23]. It has to be taken into consideration that some of the PAR agonists, such as thrombin, activate more than one receptor. Indeed, in addition to PAR1, thrombin activates PAR4 [20] and type-II TGFβ receptors and consequently leads to their down regulation [64]. PAR1 [65] and possibly PAR3 and PAR4 are involved in vascular inflammation, but the primary role for PAR2 seems to be cytoprotection in the gastro intestinal tract [66,67] and airways [68]. It was reported that whereas PAR1^-/- ^mice do not mount inflammatory responses to a variety of stimuli, PAR2^-/- ^mice present a delayed microvascular inflammation [40] and a reduced encephalomyelitis [69].

Another possible explanation for the discrepancy between PAR1 and PAR2 is the differential localization of the two PARs in urinary bladder. PAR2 is much more abundant than PAR1 in the urothelium. However, following acute and chronic inflammation, probably because of the shedding of urothelial cells bearing PARs, both PAR1 and PAR2 expression are reduced [24]. Alternatively, the association between PAR1 and PAR2 with vallinoid receptors [70] in sensory C fibers deserves further investigation as a possible explanation for a differential participation of PAR1 and PAR2 in inflammation. Another hypothesis points to the control of PAR by a metalloproteinase-dependent shedding of proteins from the cell surface [56]. More recently, it has been shown in humans that the downstream signal transduction cascade in response to PAR2 but not PAR1 depends on an interacting partner, the Jun activation domain-binding protein 1 (Jab1) [71]. Taken together, data from literature suggests a differential fate for PAR1 receptor as compared to PAR2 receptor, which could help to explain the difference in response to inflammatory stimuli in the bladder of PAR1^-/- ^versus PAR2^-/- ^mice. Regardless of the receptor type, our findings demonstrate an overwhelming participation of PARs in bladder inflammation and place them in a central role controlling the communication between the immune system represented by mast cells, the sensory system represented by SP, and infection represented by LPS.

## Conclusion

This work indicates an overriding participation of PAR1 receptors in bladder inflammation, provides a working model for the involvement of a network of transcripts downstream of PAR1 activation, and evokes testable hypotheses regarding the regulation of PAR. It remains to be determined whether PAR1 receptor blockade or selective gene silencing of transcripts downstream of PAR activation will ameliorate the clinical manifestation of cystitis. Inhibiting of PAR up-regulation using small interfering RNA technology, as confirmed by immunoblotting, should substantially reduced bladder inflammatory response as it has been shown in other systems [36].

## Methods

### Human Urothelial Cell Culture

Human bladder carcinoma cell line J82 (HTB-1) were obtained from the American Tissue Culture Collection. J82 cells were seeded onto a 4-chambers slide and cultured in Minimum Essential Media (MEM), supplemented with 10% fetal bovine serum (FBS), 100 μM non-essential amino acids, 1 mM sodium pyruvate, 100 U/ml penicillin/streptomycin and 2 × MEM Vitamin Solution. Cells were maintained at 37°C in a humidified atmosphere containing 5% CO_2 _until 90% confluence was reached. The medium was removed and cells were fixed in 10% formalin.

### PARs IHC in human cell line

Fixed cells were processed for routine immunohistochemistry according to published methods [27]. All reagent incubations (30 min) and washes were performed at room temperature. Normal blocking serum (Vector Labs, Burlingham, CA) was placed on all slides for 10 min and, after a brief rinse in PBS, sections were incubated with primary polyclonal (Santa Cruz Biotechnology, Santa Cruz, CA) antibodies: PAR-1 (1:20), PAR-2 (1:100), PAR-3 (1:5), and PAR-4 (1:50). Slides were washed and incubated with biotinylated secondary antibodies (Vector Labs), and goat anti-rabbit (polyclonal antibodies). After rinsing in PBS, the avidin-biotin-horseradish peroxidase complex reagent (ABC-HRP, Vector Labs) was added. Slides were washed and treated with the chromogen 3,3'-diaminobenzidine (DAB, Biomeda, Foster City, CA) (two changes, 5 min each), rinsed in dH_2_0, counterstained with hematoxylin, dehydrated, and coverslipped with Permount (Fisher Scientific) mounting media. Positive cells were visualized by microscope (Eclipse E600, Nikon, Lewisville, TX). All tissues were photographed at room temperature by a digital camera (DXM1200; Nikon). Five random fields per slide were counted. Images were analyzed with Image-Pro Analyzer^® ^(Media Cybernetics Inc.; Silver Spring, MD). The number of positive cells per field at 200 × magnification was calculated as percent of the total cells (200 × magnification). Results are presented as average and standard error of the mean.

### PARs Polymerase chain reaction assay (PCR) in human cell line

Total RNA was extracted in Ultraspec RNA solution (Biotecx Laboratories Inc. Houston, TX) according to the manufacturer's instructions. The amount and quality of the RNA were verified by measuring the absorbance at 260 and 280 nm, and by electrophoresing the samples on a formaldehyde/agarose gel. Oligo(dT)-primed reverse transcription of RNA was performed with the SuperScript First-Strand Synthesis System for reverse transcriptase-polymerase chain reaction (RT-PCR) (Invitrogen, Carlsbad, CA), using 5 μg of RNA for each reaction. Following reverse transcription, PCR amplifications were performed from 2 μl of each cDNA. Primer pairs were designed using Primer 3 [72]. Details of the primer sequences are given in additional file 1 (Table 1) and were designed according to reference [73]. The designed primers shared 100% homology with the target sequence but no significant homology with other sequences.

The PCR program consisted of one preincubation at 94°C for 2 min and 40 cycles at 94°C for 30 s, 55°C for 30 s (50°C for 1 min for PAR1), 68°C for 1 min (3 min for PAR1), and 68°C for 5 min. All PCR reactions were performed with a Robocycler Gradient 96 with a heated lid (Stratagene, La Jolla, CA) in 50 μl of 1 × PCR buffer, 1.5 mM MgCl2, forward and reverse primers at 0.2 μM, 200 μM dNTP, and 1 U of *Taq *DNA polymerase (Invitrogen). Twenty microliters of the amplification mixtures were analyzed by agarose gel electrophoresis. Beta-actin was used as positive control. Images of the PCR products were taken using the FluorChem HD digital darkroom (Alpha Innotech, San Leandro, CA) and the area under the curve was quantified using Image J software [74].

### Animals

All animal experimentation described here was performed in conformity with the "Guiding Principles for Research Involving Animals and Human Beings (OUHSC Animal Care & Use Committee protocol #05-088I). PAR1^-/-^[38], PAR2^-/- ^[75], and C57BL/6J mice were used in this research. C57BL/6J mice were used as wild type since PAR1-/- and PAR2-/- were enriched in this background.

### Antigen sensitization protocol

One group of mice was sensitized with 1 μg DNP4-human serum albumin (HSA) in 1 mg alum on days 0, 7, 14, and 21, intraperitoneally (i.p.). This protocol induces sustained levels of IgE antibodies up to 56 days post-sensitization [76]. One week after the last sensitization, cystitis was induced as described below.

### Induction of cystitis

Acute cystitis was induced in 8 mice per group, as described previously [27,32,48,77,78]. Briefly, female wild type (C57BL/6J), PAR1^-/-^, and PAR2^-/- ^mice were anesthetized (ketamine 200 mg/kg and xylazine 2.5 mg/kg, i.p.), then transurethrally catheterized (24 Ga.; 3/4 in; Angiocath, Becton Dickson, Sandy, Utah), and the urine was drained by applying slight digital pressure to the lower abdomen. The urinary bladders were instilled with 200 μl of one of the following substances: pyrogen-free saline, SP (10 μM), Escherichia coli LPS strain 055:B5 (Sigma, St. Louis, MO; 100 μg/ml), antigen (in sensitized mice; DNP4-OVA 1 μg/ml), control inactive peptide (LRGILS [55]), or PAR- activating peptides (PAR1-AP = SFFLRN [55]; PAR2-AP = SLIGRL [55]; and PAR4-AP = AYPGKF [79]). Substances were infused at a slow rate to avoid trauma and vesicoureteral reflux (18). To ensure consistent contact of substances with the bladder, infusion was repeated twice within a 30-min interval and a 1-ml TB syringe was kept on the catheter end to retain the intravesical solution for at least for 1 hour. The catheter was removed, and mice were allowed to void normally. Twenty-four hours after instillation, mice were euthanized with pentobarbital (200 mg/kg, i.p.), and the bladders were removed rapidly.

### Quantification of inflammation

H&E stained sections were visualized under microscope (Eclipse E600, Nikon, Lewisville, TX). All tissues were photographed at room temperature by a digital camera (DXM1200; Nikon). Exposure times were held constant when acquiring images from different groups. Images were analyzed with Image-Pro Analyzer^® ^(Media Cybernetics Inc.; Silver Spring, MD 20910). The number of polymorphonuclear [PMNs] leukocytes was counted in a blinded fashion in 10 random fields per slide in two non-consecutive sections per urinary bladder at 200 × magnification. The number of PMNs was normalized per cross-sectional area (μm^2^). The number of infiltrate PNMs was the most reproducible sign of acute bladder inflammation and, therefore, it was used for quantification.

### Statistical Analysis

Data in figures represent the mean ± SEM of the indicated number of samples. The difference between two mean values was analyzed with the unpaired Student's t-test. Since we did not assume equal variance because the variance of PMN population is unknown, p values were correct using a Welch's test (GraphPad Prism software version 4.0; GraphPad Software, Inc. San Diego, CA 92130). A value of p < 0.05 was considered statistically significant.

### Materials

PAR1-AP, PAR2-AP, and PAR4-AP were synthesized at the Molecular Biology Resource Facility, William K. Warren Medical Research Institute, OUHSC, as carboxyl-terminal amides, purified by high-pressure liquid chromatography, and characterized by mass spectroscopy. Peptide solutions were made fresh in PBS from powder.

## Competing interests

The author(s) declare that they have no competing interests.

## Authors' contributions

All authors read and approved the final manuscript. **RS **conceived of the study and drafted the manuscript, **MRD **participated in the design and reviewed the morphological results, **PAG **participated in the experimental design and provided PAR1 ^-/- ^and PAR2 ^-/- ^mice, **CKD **participated in design and proper use of PAR-APS, **ID **participated in the experimental design, **MI **participated in its design and helped to draft the manuscript, **REH **participated in its design and helped to draft the manuscript, **CS **helped **MRS **with animal experiments, **CAD **performed the experiments using human urothelial cell line (J82), and **MRS **participated in its design, carried out the animal experiments, and removed the tissues.

## Supplementary Material

Additional file 1 Primers for PCR. Table 1 Click here for file
