# Supplementary material for: Mandatory role of proteinase-activated receptor 1 in experimental bladder inflammation
Source: BMC Physiol. 2007 Mar 30;7:4. doi: 10.1186/1472-6793-7-4 (PMC1853108; doi:10.1186/1472-6793-7-4)
Supplement: Additional file 1 — Primers for PCR. Table 1 [file 1472-6793-7-4-S1.pdf]

Table 1 Primers for PCR

Gene	Forward	Reverse
PAR-1	TGTGAACTGATCATGTTTATG	TTCGTAAGATAAGAGATATGT
PAR-2	AGAAGCCTTATTGGTAAGGTT	AACATCATGACAGGTCGTGAT
PAR-3	CTGATACCTGCCATCTACCTCC	AGAAAACTGTTGCCCACACC
PAR-4	ATTACTCGGACCCGAGCC	TGTAAGGCCCACCCTTCTC
B-Actin	CCTTCCTGGGCATGGAGTCCTG	GGAGCAATGATCTTGATCTTC
